# Topological alternation from structurally adaptable to mechanically stable crosslinked polymer

**DOI:** 10.1080/14686996.2021.2025426

**Published:** 2022-02-01

**Authors:** Wei-Hsun Hu, Ta-Te Chen, Ryo Tamura, Kei Terayama, Siqian Wang, Ikumu Watanabe, Masanobu Naito

**Affiliations:** a Research and Services Division of Materials Data and Integrated System (MaDIS), National Institute for Materials Science (NIMS), Ibaraki, Japan; b Graduate School of Science and Technology, University of Tsukuba, Ibaraki, Japan; c Research Center for Structural Materials, National Institute for Materials Science, Ibaraki, Japan; d International Center for Materials Nanoarchitectonics (WPI-MANA), National Institute for Materials Science, Ibaraki, Japan; e Graduate School of Medical Life Science, Yokohama City University, Kanagawa, Japan

**Keywords:** Covalent adaptable network polymer, shape-morphing polymer material, creep deformation, topological alternation, 20 Organic and soft materials (colloids, liquid crystals, gel, polymers), 301 Chemical syntheses / processing < 300 Processing / Synthesis and Recycling, 501 Chemical analyses < 500 Characterization

## Abstract

Stimuli-responsive polymers with complicated but controllable shape-morphing behaviors are critically desirable in several engineering fields. Among the various shape-morphing materials, cross-linked polymers with exchangeable bonds in dynamic network topology can undergo on-demand geometric change via solid-state plasticity while maintaining the advantageous properties of cross-linked polymers. However, these dynamic polymers are susceptible to creep deformation that results in their dimensional instability, a highly undesirable drawback that limits their service longevity and applications. Inspired by the natural ice strategy, which realizes creep reduction using crystal structure transformation, we evaluate a dynamic cross-linked polymer with tunable creep behavior through topological alternation. This alternation mechanism uses the thermally triggered disulfide–ene reaction to convert the network topology – from dynamic to static – in a polymerized bulk material. Thus, such a dynamic polymer can exhibit topological rearrangement for thermal plasticity at 130°C to resemble typical dynamic cross-linked polymers. Following the topological alternation at 180°C, the formation of a static topology reduces creep deformation by more than 85% in the same polymer. Owing to temperature-dependent selectivity, our cross-linked polymer exhibits a shape-morphing ability while enhancing its creep resistance for dimensional stability and service longevity after sequentially topological alternation. Our design enriches the design of dynamic covalent polymers, which potentially expands their utility in fabricating geometrically sophisticated multifunctional devices.

## Graphical abstract

**Figure:**
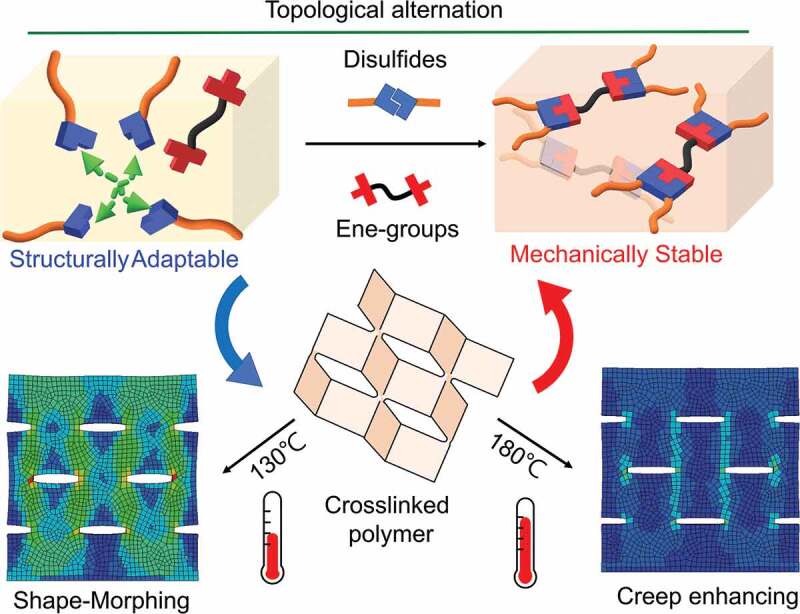

## 1. Introduction

Shape-morphing in response to the environment is ubiquitous in nature. Its diversity and the accompanied multi-functions are essential for meeting daily challenges. There is a growing interest for synthetic approaches to mimic stimuli-responsive shape-morphing behaviors with similar intelligence [1]. Remarkably, the drive to develop more disparate materials that match the complexity of the vast technological potential appears to be a never-ending task. Recent years have witnessed the fast progress of shape-malleable cross-linked polymers based on dynamic covalent bonds, a set of materials often referred to as covalent adaptable networks (CANs) [2–4]. Under specific stimuli, their network topology can rearrange through a rapid bond exchange reaction. Subsequent reversion of the stimulus returns the network polymers to their original chemically cross-linked state. Such dynamic character realizes a re-shaping capability while still retaining many of the beneficial properties of the cross-linked polymer. Their shape-morphing mechanism mainly relies on the solid-state plasticity through a bond exchange reaction instead of fluidic-state plasticity with macroscopic melting. Thus, dynamic cross-linked polymers are able to achieve a cumulative effect for complex shape change independent of molding [5–7]. Although dynamic cross-linked polymers are promising prospects, their use in practical industrial applications is still far from straightforward owing to an essential drawback of dynamic topology – creep deformation [8–11]. Creep is the tendency of a continuous and permanent deformation resulting from exposure to mechanical stress. It is highly undesirable for most engineering fields as it would negatively reduce the dimensional stability and thermomechanical performance.

There are many strategies for suppressing creep deformation while developing dynamic cross-linked polymers with excellent creep resistance and dimensional stability. For example, creep suppression could be leveraged by adjusting the activity of the exchange reaction [9] and the fraction dynamic/permanent cross-links in a polymer scaffold [10]. Other approaches have shown that the incorporation of dual dynamic cross-links, such as introducing, ionic bonds [11], hydrogen bonds [12] or orthogonal stimuli-responsive dynamic bonds [13], is beneficial because they improve creep resistance but maintain sufficient re-processibility by the second dynamic system. Alternatively, phase separation in a block copolymer uses highly ordered micro-/nano-structures to inhibit the creep deformation [14]. Current approaches introduce purposely molecular designs into cross-linked polymer at the synthesis step; thus, they are able to effectively enhance the creep resistance yet simultaneously reduce their shape reconfigurability. On the other hand, natural materials have also been able to resolve these creep issues by taking an opposite strategy. For example, glaciers and snow sliding are classic phenomena that rely on ice creep. These creep behaviors are regulated through an inner structural alternation, such as ice grain size, crystallized types, and topologies, in the crystal ice through the re-crystallization process [15–18]. Inspired by these structurally controlled strategies, synthetic analogs of these natural mechanisms provide an alternative approach to creep suppression while maintaining the dynamic features in a covalent adaptable network polymer.

Herein, we evaluate a dynamic cross-linked polymer that allows shape-morphing but subsequentially realizes creep reduction through thermally triggered topological alternation in a solid-state network. The macromolecular topological control plays a vital role in polymers as it considerably changes the material properties. However, dynamic cross-linked polymers just rearrange their topology by reshuffling the network joints which keeping a nearly constant number of cross-links. Conversely, the dynamic cross-linked network does not permit topological alternation afterward. Thus, these materials are dynamic while maintaining constant topological state and thermomechanical properties. Recent progress in topologically changeable dynamic networks has provided a new opportunity for programming functions of dynamic cross-linked polymers [19–21]. For example, we have synthesized a bio-inspired mechanical gradient polymer using network topological programming in a polymerized bulk-polymer [20]. In this study, by introducing topological programming mechanism, our resulted cross-linked polymer exhibits temperature-dependent topological rearranging/alternating. That is, at relatively low temperature, the topological rearrangement through bond exchange can be activated in dynamic topology, which results in thermal plasticity for the shape-morphing purpose. Following a higher temperature, a topological alternation – from dynamic to static state – occurs in the cross-linked network, thereby enhancing its creep resistance. Such temperature-dependent controllability resolves the problems of compromised design, desired shape-morphing, and undesired creep deformation in traditional approaches. The polymer material presented here can be applied to complicated three-dimensional (3D) kirigami manufacturing, which requires desirable shape programmability while exhibiting limited or no creep in long-term performance.

## 2. Experimental details and materials

### 2.1 Preparation of TP-Ene cross-linked polymer

Trimethylolpropane triglycidyl ether (TMPGDE, from Sigma-Aldrich), 3,3′-4-aminophenyl disulfide (AMDS, from Tokyo Chemical Industry Co., Ltd, TCI), and ethylene glycol dimethacrylate (EGDMA, from Tokyo Chemical Industry Co., Ltd, TCI) were mixed at 80°C for 0.5 h. (TMPDGE: EGDMA: AMDS = 10.0 g: 2.6 g: 6.7 g (36.1 mmol: 13.1 mmol: 26.9 mmol). A homogeneous precursor was obtained after stirring. The obtained precursor prepared was poured into poly(tetrafluoroethylene) (PTFE) molds, then thermally cured at 140°C for 2.5 h. The as-prepared cross-linked polymer is denoted TP-Ene.

### 2.2 Post-stabilized cross-linked network (TP-180)

The TP-Ene sample was heated to 180°C. After 2 h of the topological alternation through the thermally triggered disulfide–ene reaction, the sample was cooled down to room temperature. The sample after post-stabilization is denoted as TP-180.

### 2.3 Thermally triggered disulfide–ene model reaction

Diphenyl disulfide (0.87 g, 4.0 mmol, from Tokyo Chemical Industry Co., Ltd, TCI) was mixed with EGDMA (0.4 g, 2.0 mmol) in a 10-mL three-neck flask with dimethylsulfoxide (DMSO)-*d_6_*. The mixture was heated to 180°C for 3 h. The chemical structure of the static precursor was characterized with ^1^H NMR.

### 2.4 Machine learning

To estimate the creepy boundary conditions, we utilized the active learning-based method for phase diagrams [22–24]. In this method, the uncertain points in the phase diagram are selected based on the results by the machine-learning-based label propagation method and the least confident approach. Basically, since these uncertain points are distributed near the phase boundaries, we can determine the phase boundaries using machine learning. Here, we consider ‘creepy zone’ and ‘low creepy zone’, where the creepy rate is higher and lower than 0.15, respectively, as phases, and the black-box optimization method is performed for two-dimensional space between molar ratio and temperature. The candidate points are generated in the molar ratio with steps of 5% and the temperature with steps of 2°C. We obtained the ranking for uncertainty and determined the 5 points for the next experiments. Note that in the ranking, adjacent points were excluded for experiments.

### 2.5 Finite-element simulations

ABAQUS finite-element software was used to simulate the creep deformation in distinctive polymer materials. A kirigami sheet (20 × 20 × 1 mm) was used as a model shape. All the mechanical properties of the polymers (dynamic and static topologies) in the simulations were obtained from experimental measurements at 130°C (Figure S6).

### 2.6 Characterizations

^1^H-NMR spectra were acquired on a JEOL-ESC 400 (400 MHz) NMR spectrometer with the samples in DMSO-*d_6_*, with a tetramethylsilane internal standard. Solid-state ^13^C-NMR spectra were acquired with an Oxford NMR 300 spectrometer. The variation of glass transition temperature (*T_g_*) accompanied with topological alternation was monitored by using differential scanning calorimetry (DSC, Shimadzu DSC-60). Approximately 5 mg of the TP-Ene sample was placed inside an aluminum pan with a pierced lid. The samples were heated to 130, 140, and 180°C for 0, 5, 15, 30, 60, 120 min. Then, the *T_g_* of the heated samples was measured at a scanning rate of 10°C/min under a 50 mL/min nitrogen flow. Topological alternating kinetics in the solid network were monitored with Fourier-transform infrared spectroscopy (JASCO FT-IR 6100). Thermomechanical properties, including stress relaxation, creep deformation, high-temperature stress/strain curves, and cyclic stress relaxations, were determined on a rectangular sample (10 mm × 5 mm × 1 mm) with a Netzsch DMA-242E. For the stress-relaxation experiments, a 0.6% strain was applied and probing temperatures were in the range of 110–130°C. The resulting relaxation modulus was monitored over sufficient relaxing time (240 min). For creep tests, a 0.6-N stress step was applied, and temperatures were in the range of 80–130°C. The resulting elongation was monitored for 2 h. In addition, the creep tests with varied EGDMA compositions were measured at 0.6-N stress, and temperatures were in the range of 110–130°C. For high-temperature stress/strain curves, the tensile rates (0.16, 0.22, and 0.30%/min) were applied at 130°C. For cyclic stress relaxation, the applied cyclic step strain was alternatively fixed to 0.6 N (90 min) and 0 N (15 min). Three consecutive cycles were performed at 130°C.

## 3. Results and discussion

**Figure 1.**
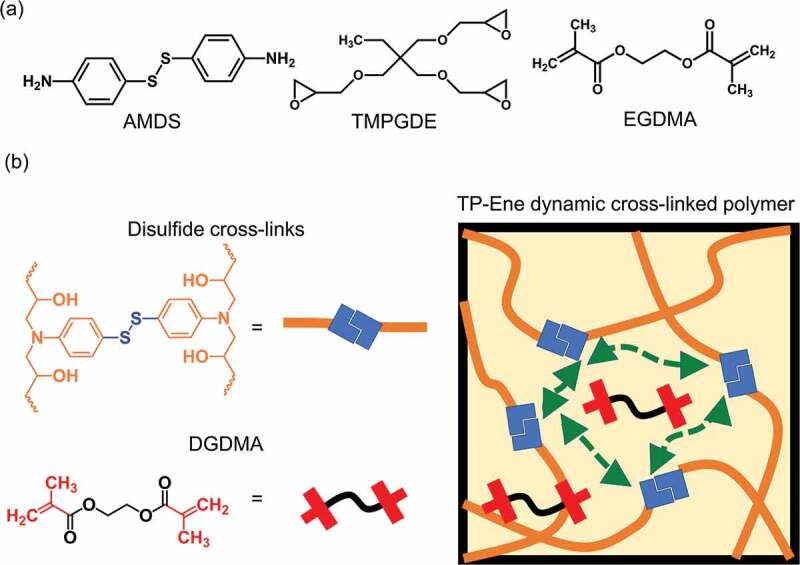
Schematic showing the design of a topologically alterable dynamic cross-linked polymer. (a) Chemical structures of starting monomers. (b) Dynamic cross-linked polymer (TP-Ene), consisting of disulfide polymer chains and EGDMA.

**Figure 2.**
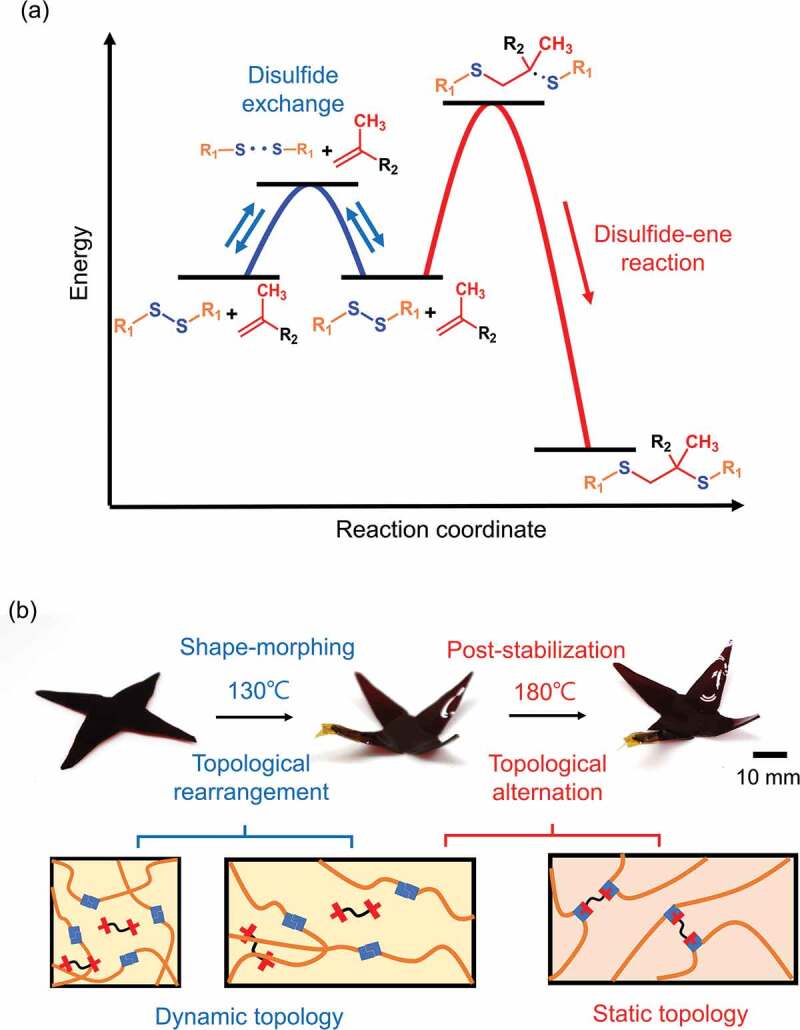
Illustration and photographs of temperature-dependent shape-morphing and post-stabilization. (a) An illustrative plot of potential energy as a function of reaction coordinate, characterized by the disulfide exchange/disulfide–ene reaction. In our system, the disulfides can undergo a reversible disulfide exchange or irreversible disulfide–ene reaction under different reaction conditions. (b) Photographs and conceptual molecular portraits of the shape-morphing and post-stabilization sequence based on two mechanisms. At 130°C, the disulfide exchange introduces topological rearrangement for shape-morphing in dynamic topology. Following the 180°C post-stabilization, the disulfide–ene reaction drives a dynamic-to-static topological alternation, which suppresses the potential creep deformation.

A disulfide/ dimethacrylate containing a cross-linked epoxy network was chosen as the model system to demonstrate the concept of a topology changeable dynamic cross-linked polymer. As shown in Figure 1(a), the network was prepared from commercially available monomers, including trimethylolpropane triglycidyl ether (TMPGDE), ethylene glycol dimethacrylate (EGDMA), and 4-aminophenyl disulfide (AMDS). The polymer scaffold was rapidly formed by thermally induced amine/epoxy polymerization. The obtained polymerized networks are denoted as TP-Ene. It is worth mentioning that the EGDMA was dissolved in the precursor but did not polymerize with the disulfides during the first curing stage. Figure 1(b) shows that the polymerized TP-Ene network consisted of inactivated EGDMA and the dynamic disulfide network. As shown in Figure 2(a), by incorporating the methacrylate groups into the disulfide network, we observed two thermally triggered reactions in our polymerized network. At different temperatures, the disulfides were capable of undergoing the reversible exchange reaction [25] or an irreversible disulfide–ene reaction [26–28]. The disulfide–ene reaction therefore offers a mechanism to alternate the molecular topology in a cross-linked polymer. Here, we define topological alternation as a dynamic-to-static transition in network topology owing to the fractional change of dynamic/permanent orthogonal cross-links. In our polymer system, the thermally triggered disulfide–ene between methacrylate and disulfide groups forms permanent cross-links with high thermodynamic stability. These permanent cross-links suppress the bond exchange reaction within the polymer network, which quenches the dynamic features; thus, the alternated topology presents a statically unrearranged characteristic. On the basis of this concept, we show a shape-morphing and post-stabilization sequence with conceptual network topologies in Figure 2(b). Upon heating to shape-morphing temperature (130°C), the rapid disulfide exchange induces topological rearrangement and simultaneously drives a viscoelastic transition with solid-state plasticity, which allows for repeated shape programmability in response to an external force. As a result, its geometry can plastically deform to particular states, then cooling fixes its dimension with robust mechanical strength. Afterward, by heating the pre-shaped object to the post-stabilization temperature (180°C), the topological alternation – from dynamic to static – can be activated in the cross-linked network as the thermally triggered disulfide–ene reaction. The static topology impedes the viscoelasticity and simultaneously enhances its creep resistance with mechanical stability. The proposed strategy provides a dynamic cross-linked polymer exhibiting proper geometric programmability while being mechanically stable for long-term performance through topological alternation in the same material.

**Figure 3.**
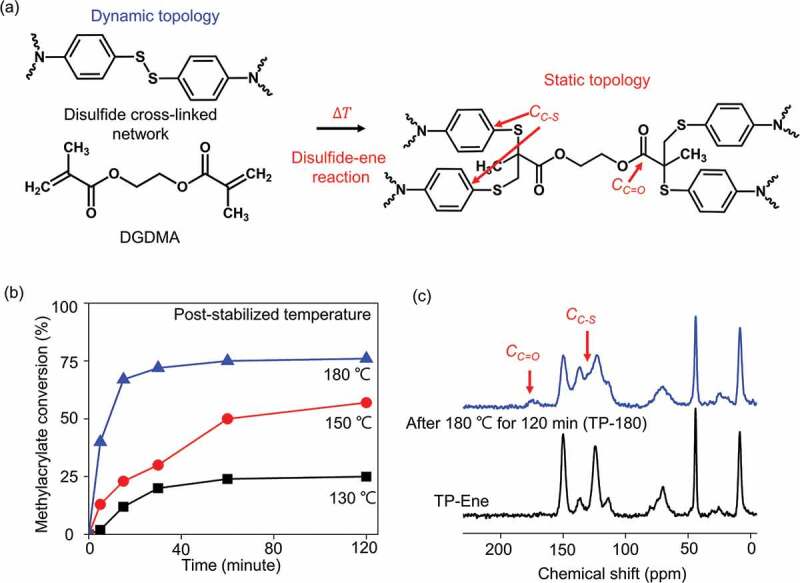
Thermally triggered topological alternation through a disulfide–ene reaction in a cross-linked polymer. (a) Network topological alternation from dynamic to static. (b) Correlation between methacrylate conversions and temperatures/times is illustrated by FTIR spectroscopy. (b) ^13^C solid-state nuclear magnetic resonance spectra of polymerized networks before (TP-Ene) and after post-stabilization at 180°C for 120 min (TP-180).

As shown in Figure 3(a), our polymer network starts with a dynamic topology. Subsequently, post-stabilization allows a static alternation in topology through the thermally triggered disulfide–ene reaction. Model experiments with proton nuclear magnetic resonance (H^1^ NMR) spectroscopy were performed to investigate the thermally triggered disulfide–ene reaction (Figure S1). The results of the model reaction between the EGDMA and diphenyl disulfide monomers indicated that disulfides reacted with methacrylate at 180°C, which yielded a permanent oligomer. This conversion implies changing from dynamic to permanent linkages within a polymer scaffold, thereby achieving topological alternation. The disulfide–ene reaction in a cross-linked polymer network was monitored with Fourier-transform infrared spectroscopy (FTIR) (Figure S2). As the above model experiments predicted, Figure 3(b) reveals that the methacrylate groups were polymerized with disulfide bonds, reaching approximately 75% conversion at 180°C for 120 min. By contrast, conversion at 130°C reached a maximum of 21% after 120 min, which suggested that the disulfide–ene reaction requires harsh conditions without catalyst [29]. The kinetics of the glass transition temperature evaluation also supported the gradual conversion observed from the FTIR results (Figure S3). The degree of methacrylate conversion in solid network polymer suggests that permanent cross-links alternate the dynamic disulfide bonds via disulfide-ene reaction. In an ideal case, the initial fraction of infeed disulfide linkages in our polymer scaffold is approximately 42.6 mol%. Afterward, the fraction of disulfide bonds, calculated by monitoring theoretical methacrylate conversion of FTIR spectra, reduces to ~10.7 mol%. This value is significantly lower than the predicted minimum fraction (40 mol%) of dynamic cross-links to keep dynamic properties in network polymer [10]. As a result, the dynamic network topology changes to the static state as the limited dynamic cross-links in the polymer scaffold. We further performed solid-state NMR spectroscopy to verify the topological alternation in the bulk-polymerized materials before and after post-stabilization at 180°C for 120 min. Figure 3(c) shows distinct network topologies for the same starting material. The new resonances that appeared after post-stabilization at 175.5 ppm and 131.3 ppm were attributed to carbonyl groups [labeled C_C=O_ in Figure 3(a)]and C-S bonds [labeled C_C-S_ in Figure 3(a)] in permanent cross-links. For simplicity, only two topological states are shown in this study: a dynamic state (TP-Ene, as-prepared) and a static state (TP-180, after 180°C post-stabilization). In reality, many intermediate states were distributed over a continuum between these two topologies (Figure S4). These spectroscopy results provided a firm verification that the thermally triggered mechanism converted from a dynamic topology into static topological states.

**Figure 4.**
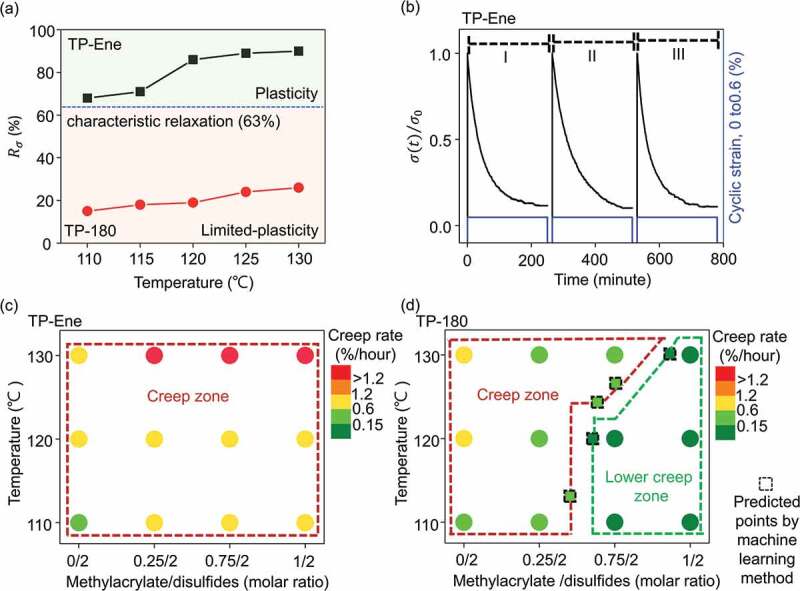
Thermally triggered topological alternating and distinct thermal plasticities. (a) Correlation of temperature-dependent stress relaxation in distinctive topological states. (b) Consecutive stress relaxation cycles of TP-Ene sample. The blue line represented cyclic strain (0 and 0.6%) at 130°C. (c) and (d) Correlation of temperature-dependent creep rates with various methacrylate/disulfide compositions. Panel (c) shows the samples without stabilized polymer (TP-Ene) and panel (d) shows the samples after post-stabilization (TP-180). Dash square represents the prediction via machine learning method.

Having established the basis of the thermally triggered topological alternation in the TP-Ene polymer, we evaluated its thermomechanical properties corresponding to distinct topologies. Their thermal plasticities were first investigated through stress relaxation experiments, typically used to quantify solid-state plasticity in dynamic cross-linked polymers (Figure S5). Here, we define a parameter of characteristic stress relaxation $R_{c}=\left(1-\sigma \left(t\right)/\sigma_{0}\right)\times 100\%$, where $\sigma \left(t\right)$ is the stress relaxation at a specific time and $\sigma_{0}$ is the initial stress. Based on the Maxwell relation, effective characteristic stress relaxation in dynamic polymers requires at least 63% stress relaxation from its initial stress within a reasonable time. For comparison, all $R_{c}$ values were obtained at the relaxation time of 230 minutes. Figure 4(a) shows the correlation of the characteristic relaxations and distinct topological states ranging from 110 to 130°C. The TP-Ene sample represented a maximum 90% of substantial stress relaxation at 130°C, which indicated cumulative plasticity in a cross-linked polymer. Therefore, we used 130°C as an appropriate temperature for shape-morphing because it achieved considerable plasticity with a minimal risk of activating sequential topological alternation. By contrast, the TP-180 sample exhibited slight relaxations (less than 33%) at varying temperatures. These differences in stress relaxations mainly resulted from the elastic and viscoelastic responses in the distinctive topological states (Figure S6). Moreover, in our system, the thermal plasticity is for shape-manipulation purposes; thus, accessing a reliable cumulative plasticity effect requires that the dynamic polymer undergo multiple cycles of stress relaxation without deterioration in its performance. Three consecutive cyclic stress relaxation experiments on TP-Ene were performed with periodic cyclic strains (0 and 0.6%) at 130°C. Figure 4(b) shows that, within each relaxation cycle, the stress repeatedly relaxed to 0.11, and no noticeable deterioration was observed upon cycling. Accordingly, these results suggested that the TP-Ene polymer has a steady and robust plasticity at 130°C without significant negative influences from the lateral topological alternation.

The above experiments demonstrated the basis of thermal plasticity in a cross-linked polymer through an exchange reaction within the dynamic topology. The sequential topological alteration offers a controllable mechanism for creep impedance in the same material. The creep behaviors were further probed at temperatures ranging from 80 to 130°C. When at a temperature above 110°C, the TP-Ene sample could not recover its initial length after stress removal (Figure S7). The unrecovered strain implied a tendency of permanent creep deformation in the cross-linking. We therefore defined 110°C as the minimized creep temperature. Furthermore, the temperature-varied creep tests were performed on the TP-Ene and TP-180 polymers with different ratios of EGDMA to systematically investigate the relationship between creep and topologies (Figure S8). In particular, these samples were prepared with the following molar ratios of 0: 2, 0.25: 2, 0.75: 2, and 1: 2 for the stoichiometric mixtures of methacrylate to disulfide functional groups. In addition, the creep behaviors were described using a steady-strain rate, $\dot{\varepsilon }=d\varepsilon \left(t\right)/dt$, where $\varepsilon \left(t\right)$ is the time-dependent strain under a certain applied stress load, and all creep rates are summarized in Table S1. In this study, the minimum creep rate was defined at 0.15%/hour, which meant the creep deformation was negligible once the strain rate was below this value. As shown in Figure 4(c), the TP-Ene samples underwent significant creep deformation; all creep rates were over 0.15% /hour, regardless of the molar ratios. Moreover, an increment of creep rate was observed with the increase of methacrylate (EGDMA monomer). This gradual growth was presumably attributed to the unreacted DGDMA within the polymer network accelerating the plastic flow arising from the defect effect [30]. The above experiments disclosed that the TP-Ene polymer exhibits poor creep resistance over a wide range of performing temperatures. Then, the following topological alternation allows further creep reduction while being made from the same starting material. Figure 4(d) demonstrates the creep rates in the TP-180 samples with different EGDMA ratios. First, the control sample without EGDMA exhibited similar creep behavior as the original because of the statistically identical topology throughout the disulfide exchange. By contrast, the creep rates were reduced significantly with increasing methacrylate. Specially, more than 85% reduction of the creep rate can be achieved when their theoretically permanent cross-links above 56 mol% in the network topology. Conversely, the permanent cross-links in topology significantly suppressed the viscoelasticity in static topology, and the corresponding creep deformation. As a result, the thermally triggered topological alternation allowed for an effective strategy for creep resistance enhancement. Furthermore, to efficiently construct a reliable creep diagram for topologically alterable polymer design, we further used machine learning to estimate the creep boundary conditions of the variation of methacrylate groups and creep temperatures [22–24] (Figure S9 and Table S2). As shown in Figure 4(d), the complicated boundary between creep and lower creep zone could be adequately plotted even when knowing only the small datasets in the initial step. This predicted diagram provides numerous underlying information for topological controlled creep behaviors. Evidently, approaching by active machine learning accelerates the design and advancement of highly functional materials with topological altering strategies.

**Figure 5.**
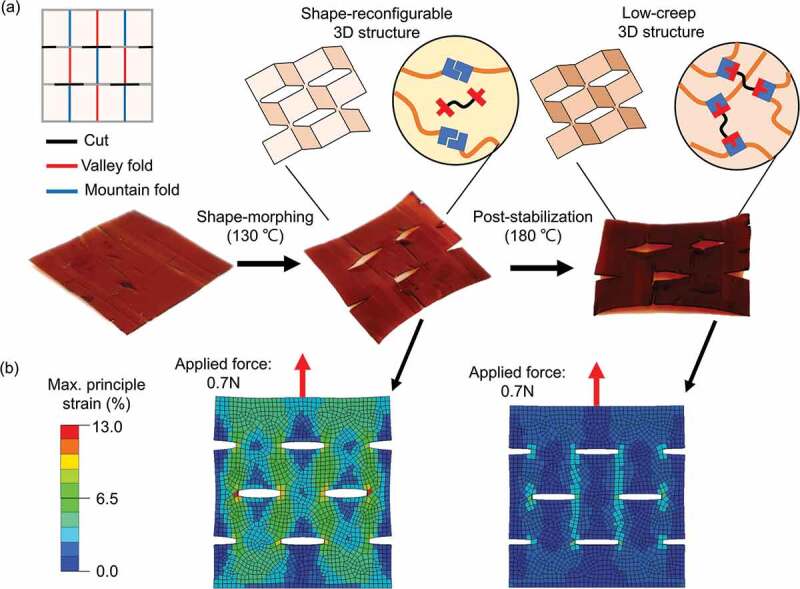
Topological alternating cross-linked polymer toward kirigami fabrication. (a) The front and side views of the 3D kirigami structure are made from a 2D polymer sheet. The 2D polymer plane can be programmed into a 3D structure through shape-morphing at 130°C plastically. Following post-stabilization at 180°C, its dimensional stability enhances owing to the topological alternation – from dynamic to static state. (b) Corresponding finite-element analysis for a kirigami structure with distinctive topologies. The color represents the magnitude of the maximum principal strain (%). The dynamic object has a high creep stain, yet the static one has a much lower strain under an identical loading condition.

Taking advantage of the temperature-dependent topologically rearranging /alternating features, our polymer material was fully compatible with a versatile method to make complicated 3D structures by kirigami technology [31–34]. The images in Figure 5 demonstrate a shape-morphing and post-stabilization sequence for kirigami manufacturing. As seen in Figure 5(a), within the shape-morphing process (at 130°C), by folding along the mountain and valley lines, the perforated polymer plane was able to transform into a 3D structure with satellite square facets and opened edges on pre-cut lines. Following post-stabilization at 180°C, the topological alternation in the network impeded potential creeping for long-term service life. We further conducted finite-element analysis to estimate the strain distribution in the kirigami structures before/after post-stabilization (Figure S10). Figure 4(b) shows the strain distribution under a constant 0.7 N of force loading. Before stabilization, a higher strain distribution was observed in the kirigami structure, while the stabilized structure had a much lower strain under identical stress. These simulations exploited that the polymeric object exhibits excellent creep resistance after post-stabilization. Although we have only demonstrated a conceptual kirigami here, incorporating proper patterns would enable more sophisticated and multifunctional 3D structures.

## 4. Conclusions

In summary, we developed a molecular design concept for a dynamic covalent network polymer that allows topological rearrangement or alternation based on temperature selectivity. The former introduces plasticity responsible for shape-morphing, and the latter contributes to an opposite effect for creep enhancement. Despite both being triggered thermally, they can be activated distinctively without overlap. The cross-linked polymer permits a versatile strategy for 3D polymer manipulation while ensuring its dimensional stability for longevity. The topologically alternating mechanism behind our system can serve as a model system to extend the design space and resolve the dilemma of current covalent adaptable network polymers to meet various practical applications.

## Supplementary Material

Supplemental Material
