# Supplementary material for: Topological alternation from structurally adaptable to mechanically stable crosslinked polymer
Source: Sci Technol Adv Mater. 2022 Feb 1;23(1):66–75. doi: 10.1080/14686996.2021.2025426 (PMC8812728; doi:10.1080/14686996.2021.2025426)
Supplement: Supplemental Material [file TSTA_A_2025426_SM0642.docx]

**Topological Alternation from Structurally Adaptable to Mechanically Stable Crosslinked Polymer**

Wei-Hsun Hu ^1,3^, Ta-Te Chen ^2,3^, Ryo Tamura ^4^, Kei Terayama ^5^, Siqian Wang ^1^, Ikumu Watanabe ^2,3^, Masanobu Naito^1,3^*

1. Research and Services Division of Materials Data and Integrated System (MaDIS), National Institute for Materials Science (NIMS), 1-2-1, Sengen, Tsukuba, Ibaraki, 305-0047, Japan.
2. Research Center for Structural Materials, National Institute for Materials Science, 1-2-1 Sengen, Tsukuba, Ibaraki 305-0047, Japan.
3. Graduate School of Science and Technology, University of Tsukuba, Ibaraki 305-8577, Japan.
4. International Center for Materials Nanoarchitectonics (WPI-MANA), National Institute for Materials Science, 1-1 Namiki, Tsukuba, Ibaraki 305-0044, Japan.
5. Graduate School of Medical Life Science, Yokohama City University, 1-7-29, Suehiro-cho, Tsurumi-ku, Kanagawa 230-0045, Japan.

**
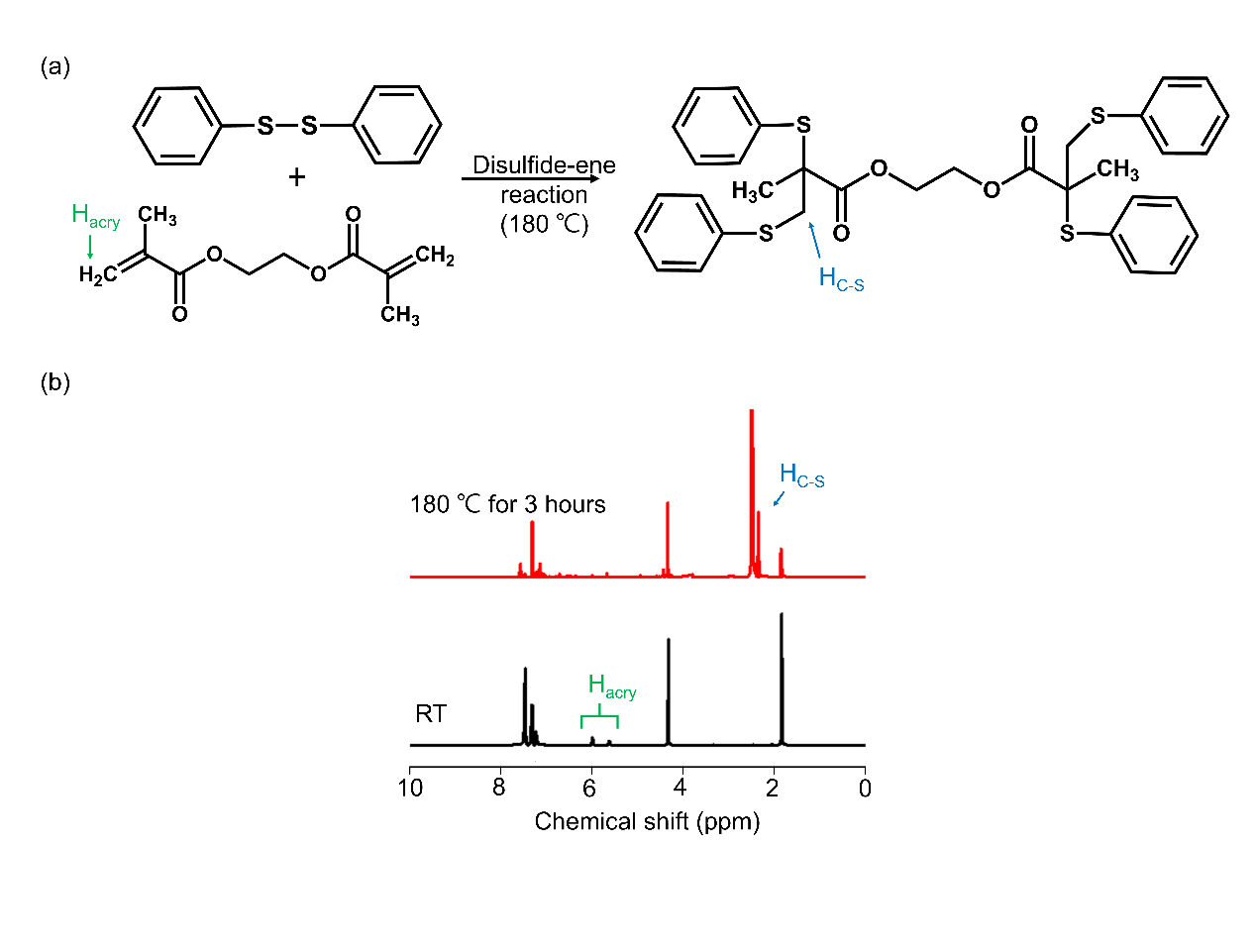
Figure S1.** Disulfide-ene model reaction. (a) Modeling compounds, EGDMA and diphenyl disulfide. (b) After 180 ℃ heating treatment, we observed that the peaks attributable to methacrylate groups at 5.62 ppm and 5.99 ppm (labeled H_acry_) gradually disappeared, and a new rise attributable to the intramolecular hemithioacetal linkages simultaneously formed at 2.35 ppm (labeled H_c-s_). The conversion implies a decline of dynamic cross-links in network topology via thermally triggered disulfide-ene reaction. We noted that the degree of methacrylate conversion in a model reaction could not reach 100% conversion. This is presumably because of the undesired side reaction, yielding the trisulfide side product [1]. During the reaction, the thiyl radical not only occurs in a disulfide-ene reaction with methacrylate groups but also simultaneously reacts with another thiyl radical or disulfide bonds.

**Figure S2.** The kinetics of solid-topological alternation via disulfide-ene reaction. (a) FT-IR spectra of TP-Ene after heating at various temperatures. (2 hours treated). (b) to (d) FR-IR spectra of TP-Ene polymer with different heating times at different temperatures. The C=C represents methacrylate units (at 1630 cm^-1^), and the original C=O at 1716 cm^-1^ represents the carbonyl group in EGDMA. During the heating process, the intensity of C=C decreased, and the C=O simultaneously shifted to a higher wavenumber, i.e. from 1716 to 1730 cm^-1^. These results of spectra strongly agreed with the topological alternation in a solid polymer network.
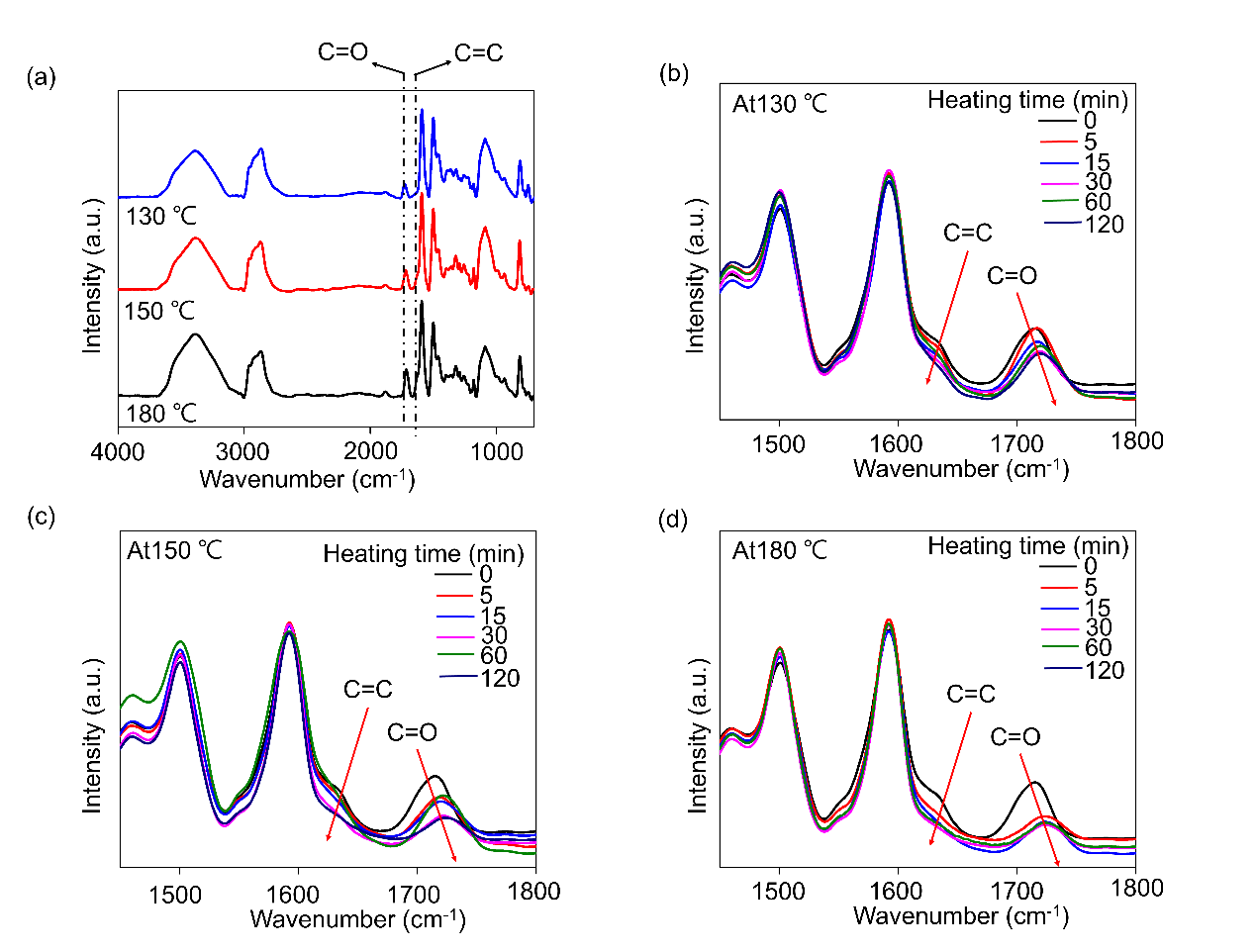


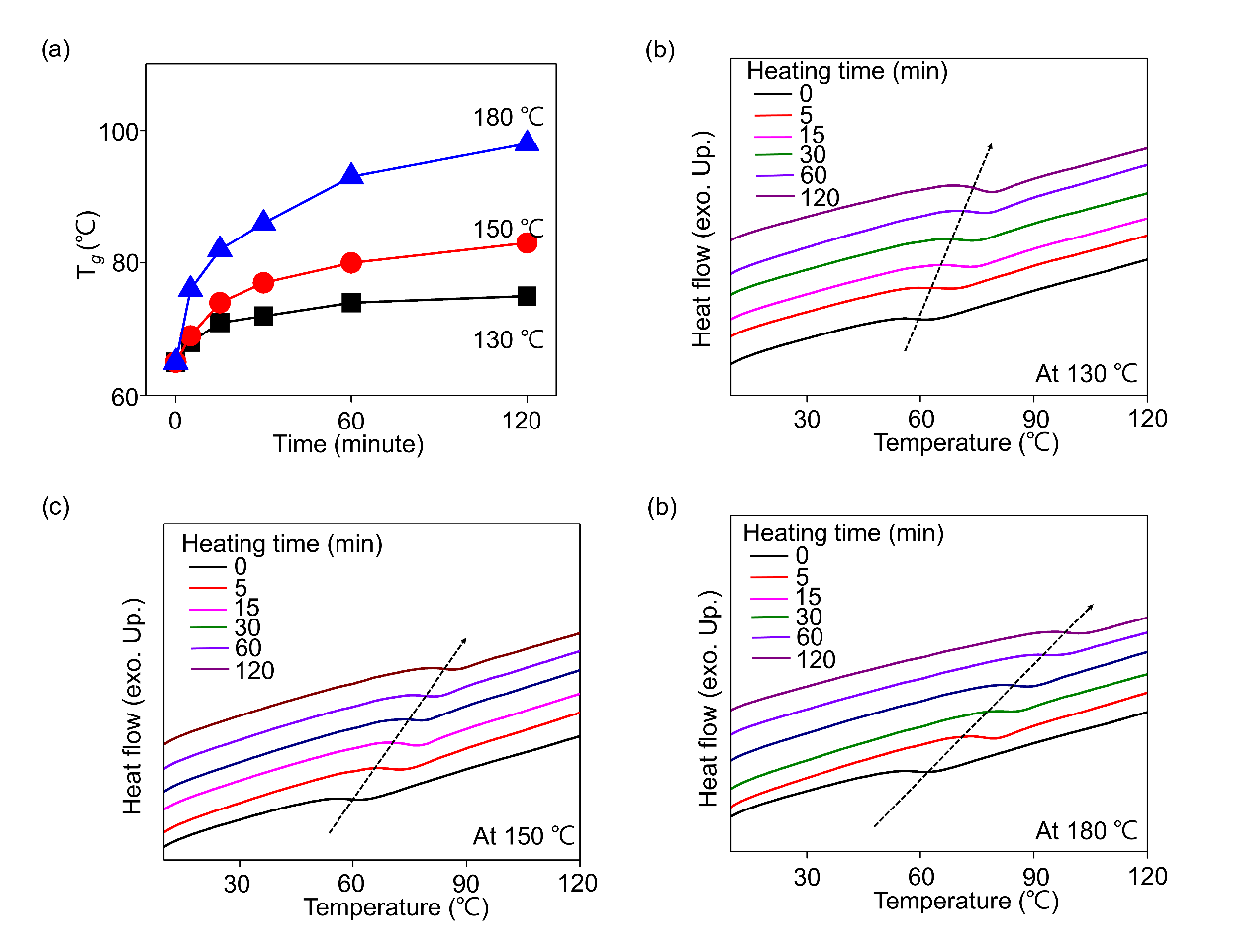


**Figure S3.** The change of glass transition temperature (T*_g_*) during topological alternation. (a) The correlation of T*_g_* under various heating temperatures and times. (b) to (d) the DSC curves of TP-Ene under different post-stabilized temperatures. The evaluation of T*_g_* illustrated the network topological change with corresponding with cross-linked density increasing in a polymerized material.

**
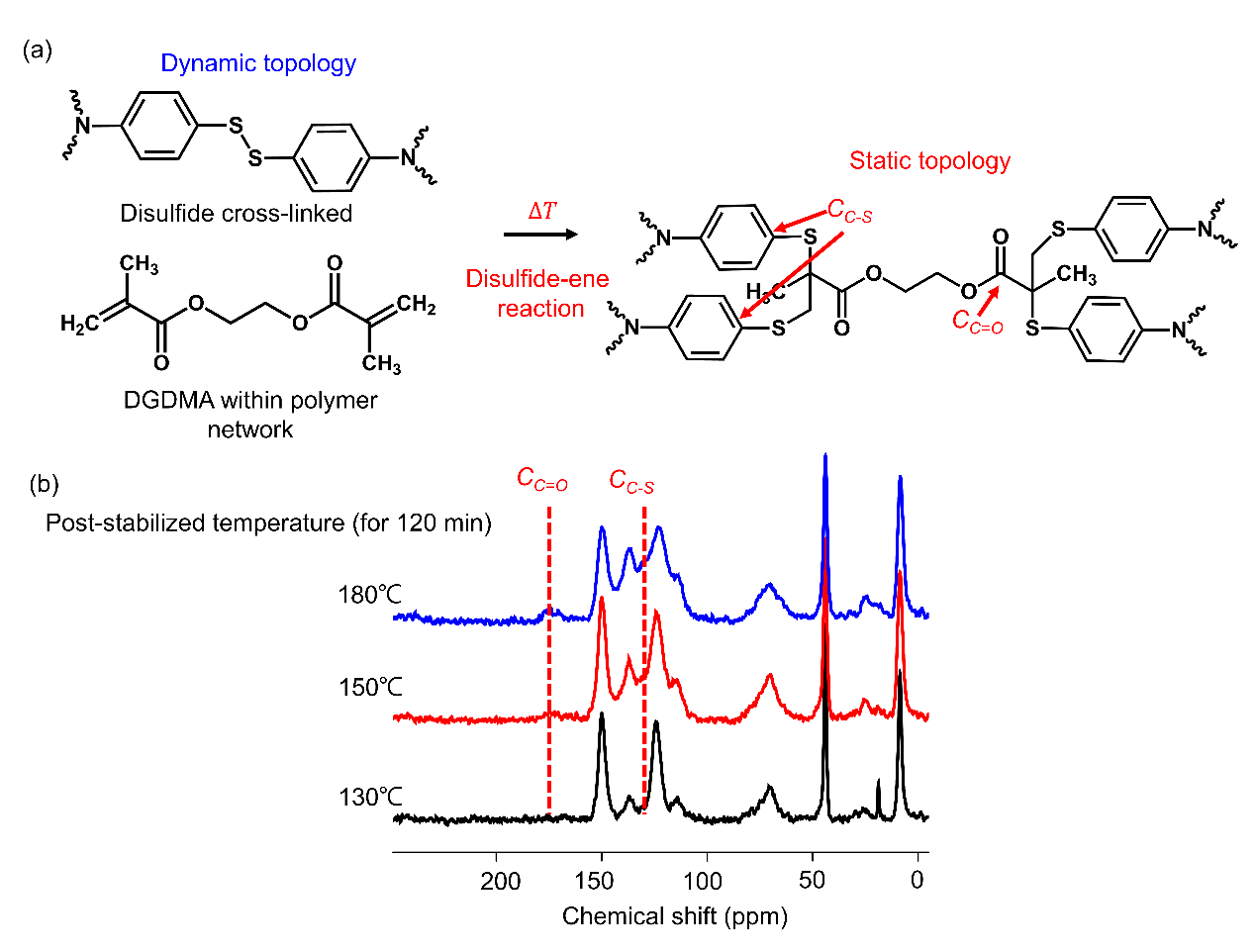
Figure S4. Topological alternation in the solid-state network. (a)** Network topological alternation from dynamic to static. (b) ^13^C solid-state nuclear magnetic resonance spectra of polymerized networks at different post-stabilized temperatures, 130, 150, and 180 ℃ for 120 min, respectively. These spectra results indicated that many intermediate topological states were distributed on a continuum between these two topologies.

**
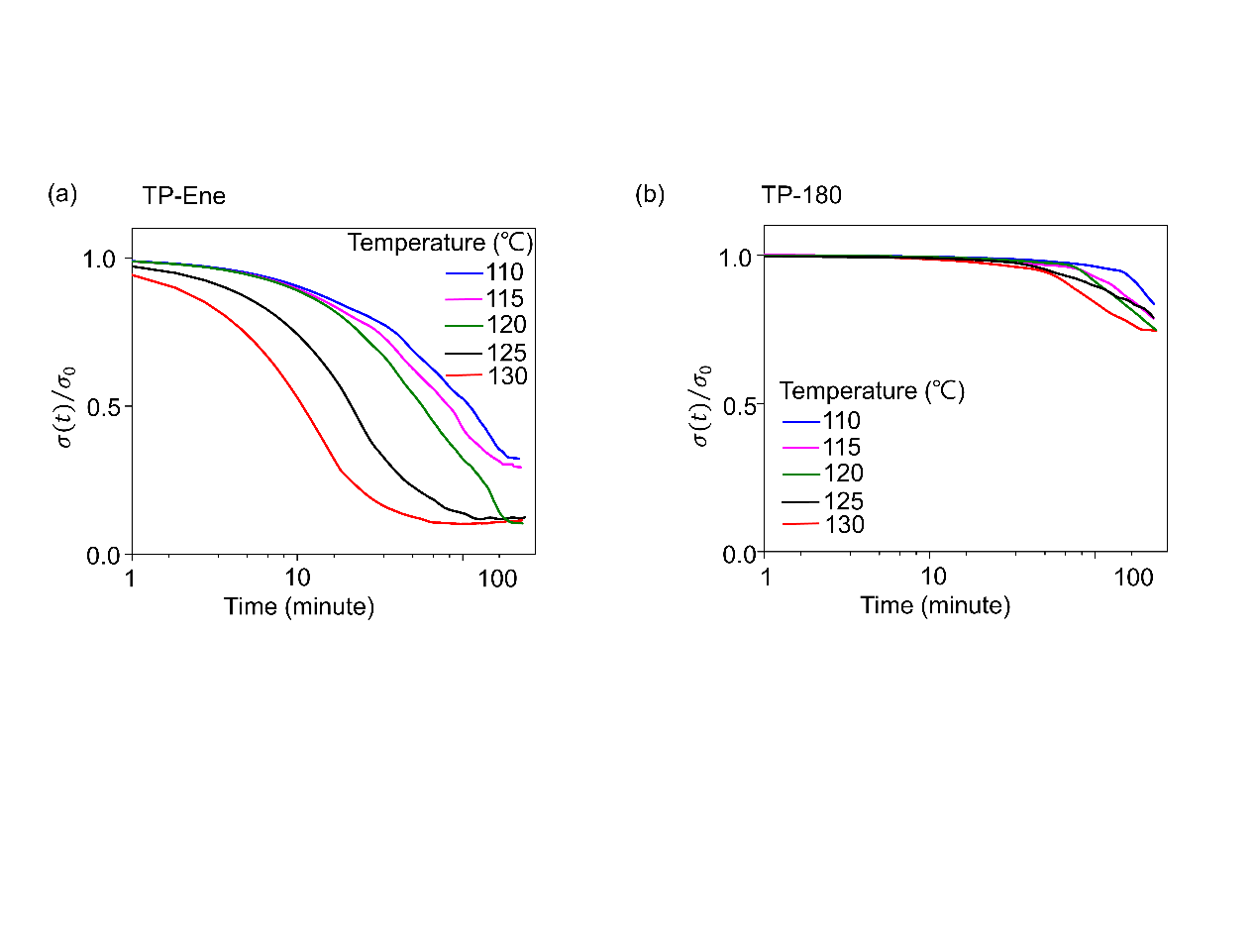
Figure S5.** Stress relaxation experiments. (a) Stress relaxation of TP-Ene (dynamic topology) ranged from 110 to 130 ℃. (b) Stress relaxation of TP-180 (static topology) ranged from 110 to 130 ℃. The TP-CSC sample could not relax to 63% of the initial stress, that is a characteristic stress relaxation in covalent adaptable network, within the experimental time scale. On the other hand, the TP-Ene polymer represents substantial stress relaxations, suggesting the TP-Ene sample exhibits viscoelastic transition in dynamic topology.

**
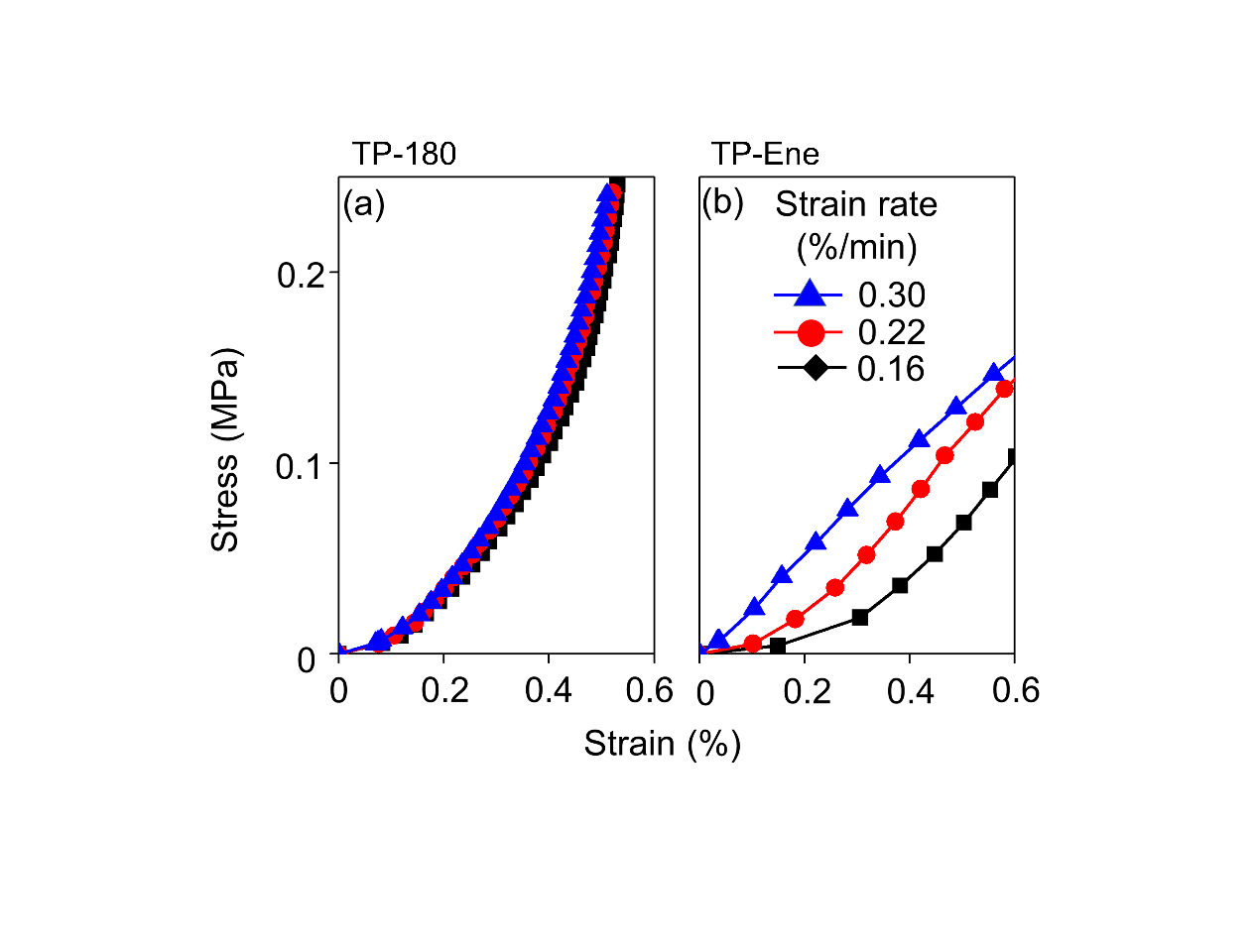
Figure S6.** Strain-rate dependent tensile test at 130℃. (a) Strain rate-independent behavior in TP-180 sample. (b) Strain rate-dependent behaviors in TP-Ene sample. The strain rate-dependent property reveals the flow state due to bond exchange in dynamic topology, resulting in viscoelasticity in cross-linked polymer (TP-Ene). Whereas the limited exchange reaction in static topology does not allow to drive the sufficient rearrangement. Thus, TP-180 shows a typical elastic response, independence of strain rates. These differences among elastic/viscoelastic responses reflected thermally triggered plasticities between dynamic/ static topologies.

**
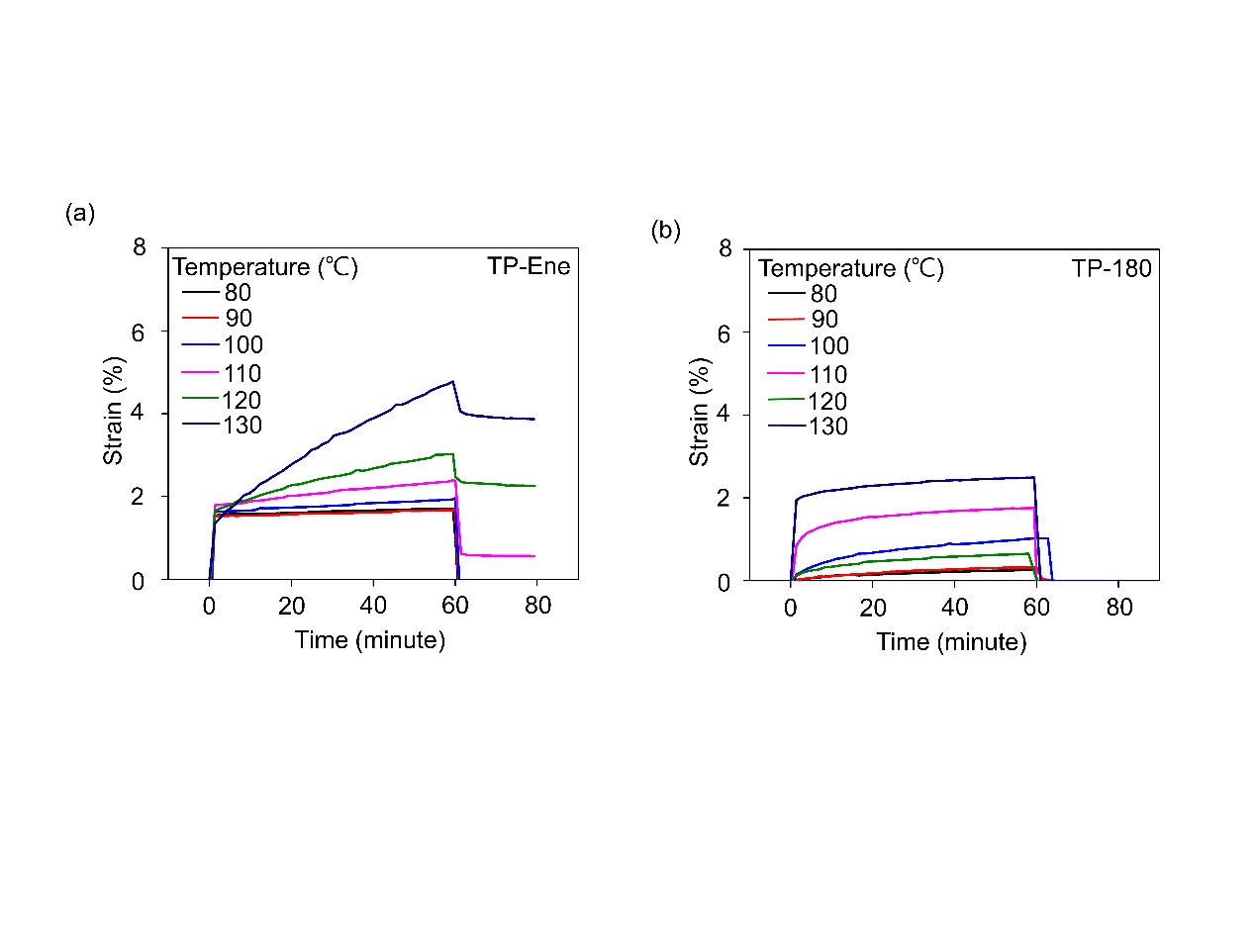
Figure S7.** Creep at various temperatures. (a) In TP-Ene polymer, an occurrence of unrecoverable strain, for example, the samples retained 20% elongation after stress removed when temperature above 110℃. (b) The length of the TP-180 samples almost totally recovered to its original state, suggesting excellent creep resistance.

**
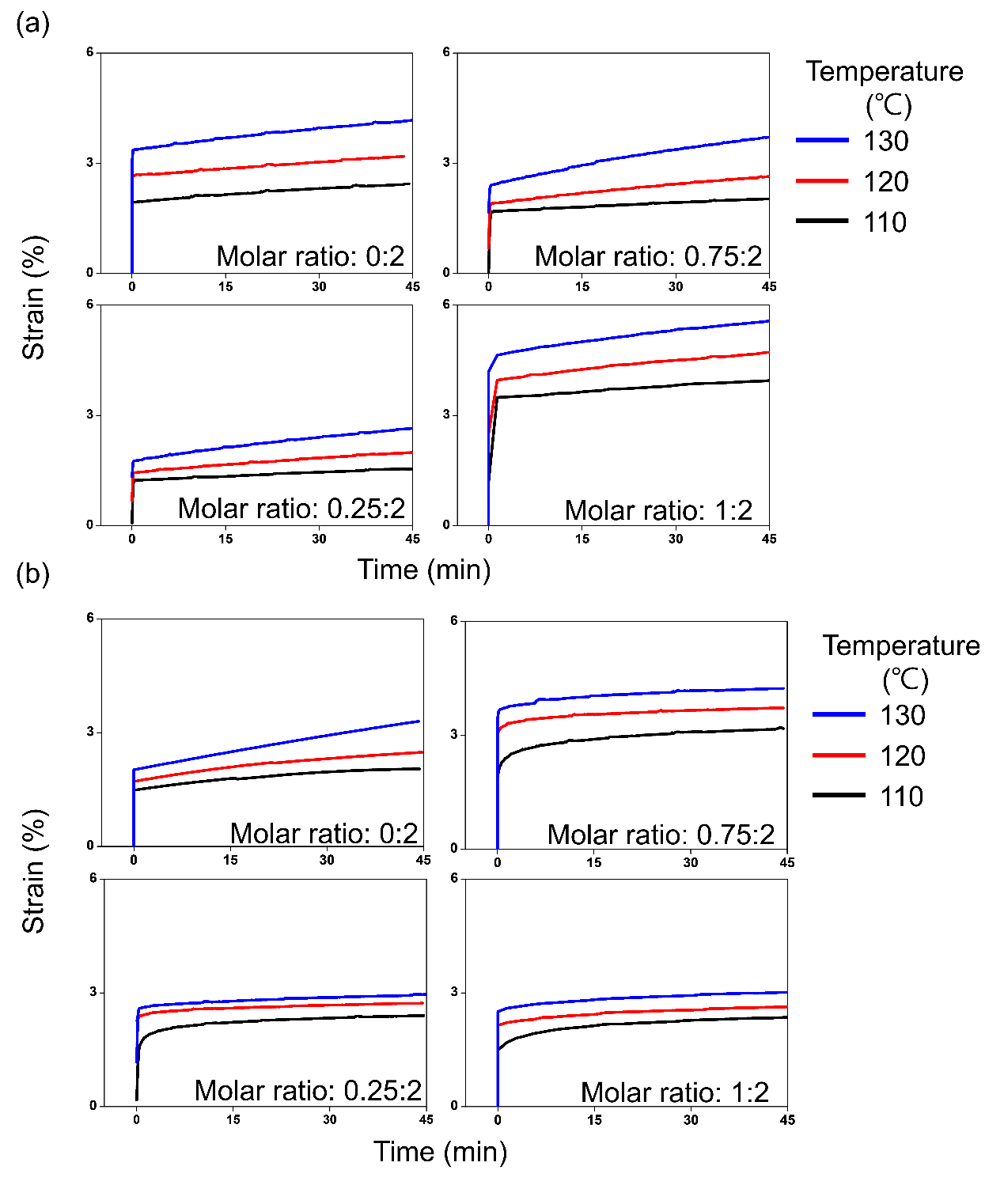
Figure S8.** Creep tests between TP-Ene and TP-180 samples with various methacrylate to disulfide molar ratios, 0:2, 0.25:2, 0.75:2, and 1:2, respectively. (a) TP-Ene samples. (b) TP-180 samples. The variation of creep behaviors with different molar ratios suggested the gradual transition in network topologies.

**
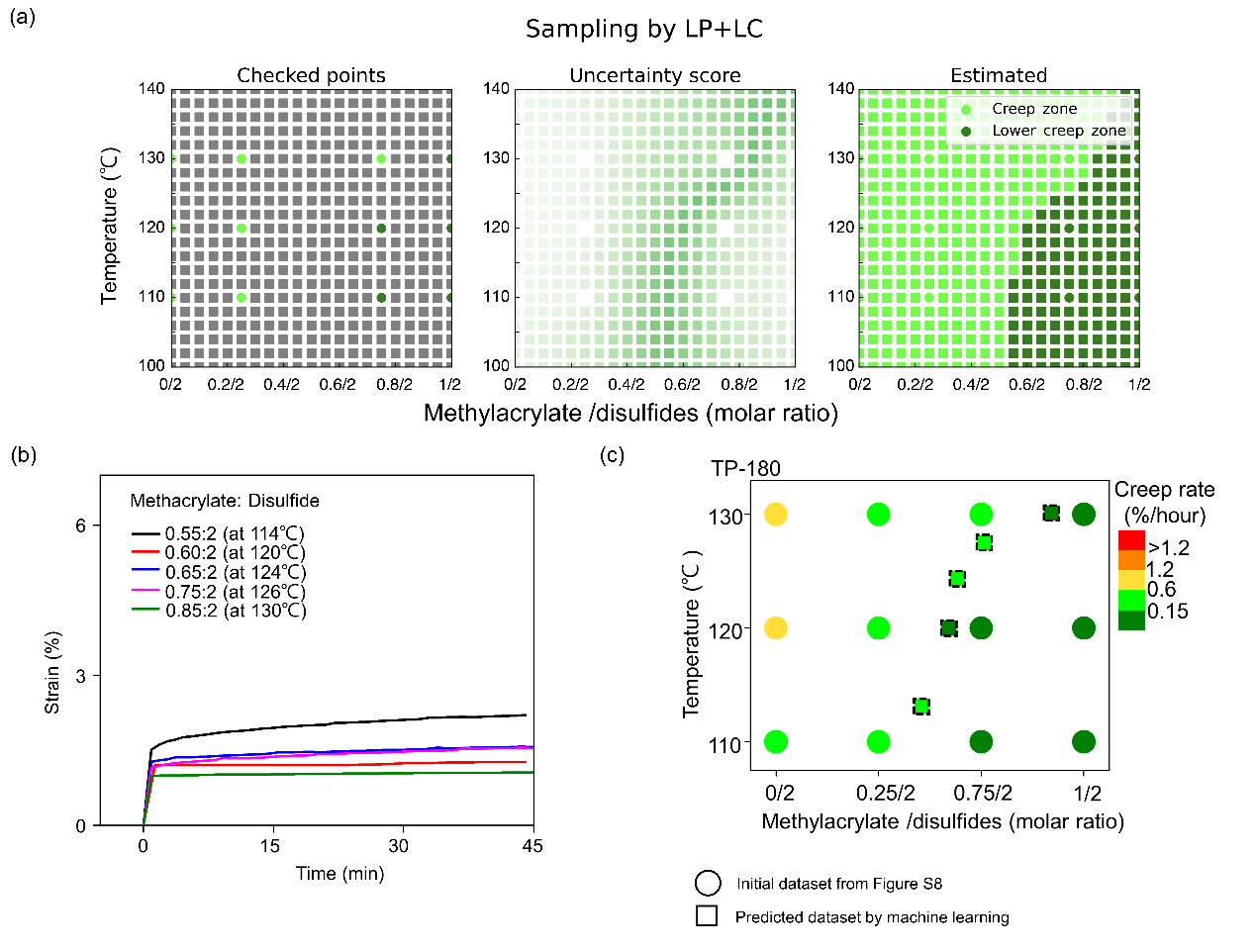
Figure S9.** The estimation of boundary conditions in creep behaviors through active learning from a small dataset. (a)Illustration of the machine learning approach. (b) Experimental results based on machine learning predications. (c) Distribution of creep behaviors with varied temperatures and methacrylate /disulfide molar ratios. The initial dataset from Figure S8 and Table S1.

**
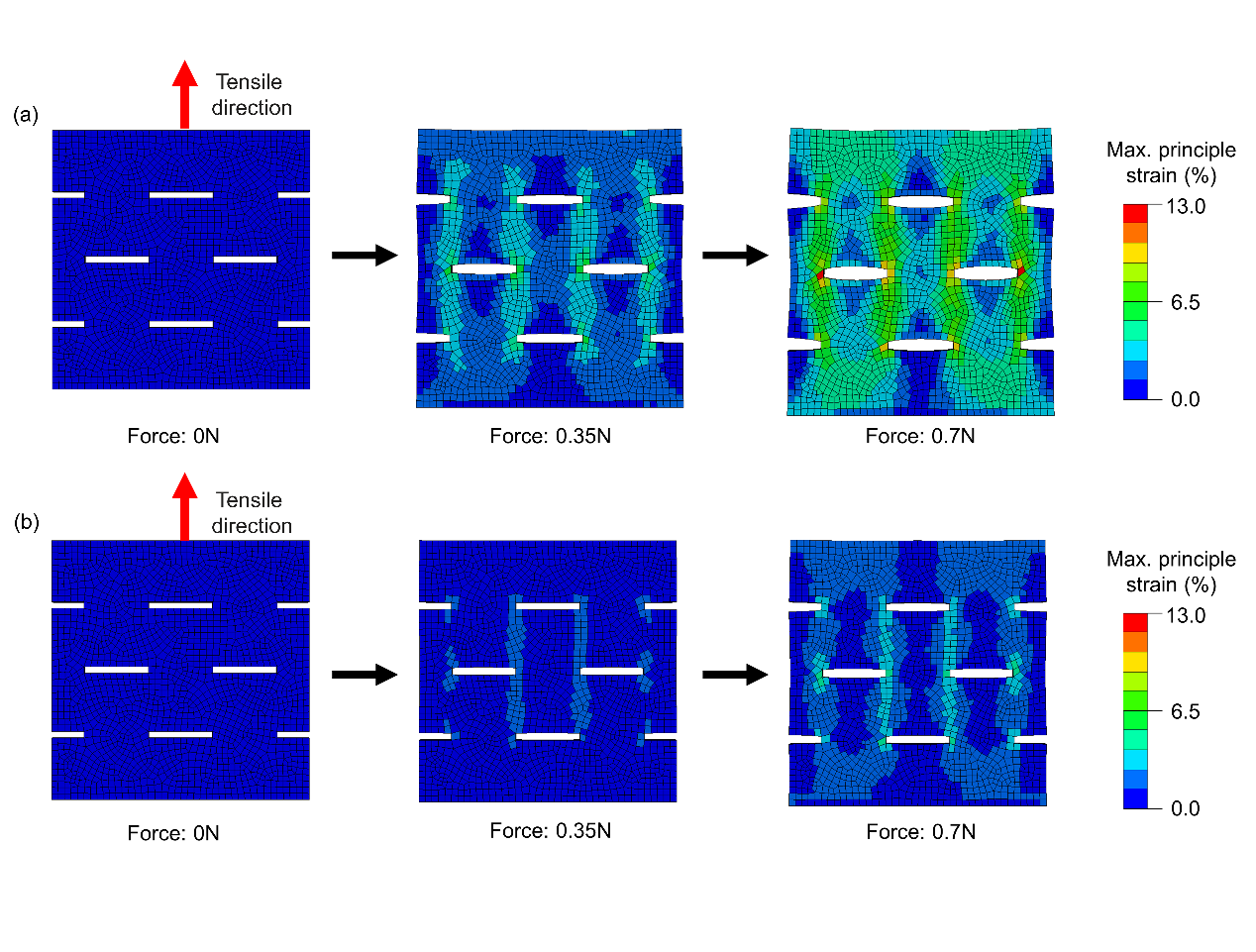
Figure S10.** Finite-element analysis of creep deformation in kirigami. (a) kirigami involved dynamic topology. (b) kirigami involved static topology. Under the same loading condition, dynamic kirigami showed a high strain distribution, yet the static one had a much lower strain distribution. These simulations were set to the experimentally measured data obtained by the stress/strain tests at 130 ℃ (Figure S5).

Table S1. Summarized creep rates (%/hour).

TP-Ene

Creep temperature

Creep rate

|  | Molar ratio of methacrylate / disulfide groups | | | |
| --- | --- | --- | --- | --- |
|  | 0:2 | 0.25:2 | 0.75:2 | 1:2 |
| 110 ℃ | 0.55±0.06 | 0.60±0.05 | 0.63±0.06 | 0.68±0.08 |
| 120 ℃ | 0.71±0.05 | 0.98±0.07 | 1.05±0.08 | 0.98±0.11 |
| 130 ℃ | 1.06±0.08 | 1.74±0.12 | 1.6±0.14 | 1.25±0.11 |

TP-180

Creep temperature

Creep rate

|  | Molar ratio of methacrylate / disulfide groups | | | |
| --- | --- | --- | --- | --- |
|  | 0:2 | 0.25:2 | 0.75:2 | 1:2 |
| 110 ℃ | 0.55±0.06 | 0.22±0.02 | 0.12±0.01 | 0.12±0.01 |
| 120 ℃ | 0.71±0.05 | 0.24±0.03 | 0.10±0.01 | 0.10±0.01 |
| 130 ℃ | 1.06±0.08 | 0.20±0.02 | 0.16±0.02 | 0.13±0.02 |

**Table S2**. Summarized experimental creep rates based on machine learning predations.

| **Methacrylate / disulfide**  **Molar ratio** | **Creep Temperatures**  **(℃)** | **Creep rate**  **(%/hour)** |
| --- | --- | --- |
| 0.55/2 | 114 | 0.18±0.02 |
| 0.60/2 | 120 | 0.11±0.01 |
| 0.65/2 | 125 | 0.21±0.03 |
| 0.75/2 | 126 | 0.21±0.02 |
| 0.85/2 | 130 | 0.13±0.01 |

References

[1] Teders M, Henkel C, Anhäuser L, Strieth-Kalthoff F, Gómez-Suárez A, Kleinmans R, Kahnt A, Rentmeister A, Guldi D and Glorius F 2018 The energy-transfer-enabled biocompatible disulfide–ene reaction *Nat. Chem.* **10** 981–8
